# First-decade patient with colorectal cancer carrying both germline and somatic mutations in *APC* gene

**DOI:** 10.1186/s12885-017-3878-0

**Published:** 2017-12-14

**Authors:** Yung-Sung Yeh, Yu-Tang Chang, Cheng-Jen Ma, Ching-Wen Huang, Hsiang-Lin Tsai, Yi-Ting Chen, Jaw-Yuan Wang

**Affiliations:** 1 Division of Trauma and Surgical Critical Care, Department of Surgery, Kaohsiung Medical University Hospital, Kaohsiung Medical University, Kaohsiung, Taiwan; 2 Department of Emergency Medicine, Kaohsiung Medical University Hospital, Kaohsiung Medical University, Kaohsiung, Taiwan; 3 Division of Colorectal Surgery, Department of Surgery, Kaohsiung Medical University Hospital, Kaohsiung Medical University, 100 Tzyou 1st Road, San-Ming District, Kaohsiung, 807 Taiwan; 4 Division of General and Digestive Surgery, Department of Surgery, Kaohsiung Medical University Hospital, Kaohsiung Medical University, Kaohsiung, Taiwan; 5 Graduate Institute of Clinical Medicine, College of Medicine, Kaohsiung Medical University, Kaohsiung, Taiwan; 6 Department of Surgery, Faculty of Medicine, College of Medicine, Kaohsiung Medical University, Kaohsiung, Taiwan; 7 Division of Pediatric Surgery, Department of Surgery, Kaohsiung Medical University Hospital, Kaohsiung Medical University, Kaohsiung, Taiwan; 8 Division of General Surgery Medicine, Department of Surgery, Kaohsiung Medical University Hospital, Kaohsiung, Taiwan; 9 Department of Pathology, Kaohsiung Medical University Hospital, Kaohsiung Medical University, Kaohsiung, Taiwan; 10 Center for Biomarkers and Biotech Drugs, Kaohsiung Medical University, Kaohsiung, Taiwan; 11 Research Center for Environmental Medicine, Kaohsiung Medical University, Kaohsiung, Taiwan; 12 Research Center for Natural products and Drug Development, Kaohsiung Medical University, Kaohsiung, Taiwan

**Keywords:** First decade, Colorectal cancer, Both germline and somatic mutations, *APC* gene, Case report

## Abstract

**Background:**

Colorectal carcinoma (CRC) is one of the most common causes of cancer-related deaths. The mean age of patients with CRC ranges from 49 to 60 years. Pediatric CRC is unusual, which often escapes early diagnosis because of a lack of awareness of its occurrence in children. The association between the mutation of *APC* and the occurrence of CRC in the first decade of life remains unknown.

**Case presentation:**

We report a 10-year-old child with CRC; he was diagnosed with stage IIIB advanced transverse colon cancer without distal metastases. We detected a heterozygous germline mutation at c.5465 T > A in both blood and tissue samples and a heterozygous somatic mutation at c.7397C > T in the tissue sample. Both of these mutations can cause CRC tumorigenesis in the first decade of life.

**Conclusions:**

The rare genetic features of this 10-year-old patient might be the predisposing cause of pediatric CRC. Therefore, screening patients with early-onset CRC through clinical and genetic characterizations is suggested.

## Background

Although colorectal carcinoma (CRC) is one of the most common malignancies in adults, it is extremely rare in children. Moreover, most of the reported cases of CRC involve older adolescents, whereas prepubertal cases are exceedingly unusual. Because of its rarity, early diagnosis and clinical management and treatment protocols are generally extrapolated from the experiences of only adults [1]. Although pediatric textbooks describe CRC, the provided information is insufficient. A frequency of 1.3 cases per one million people aged younger than 20 years has been reported, and the exact incidence rate of pediatric CRC remains unknown. In general, the younger the patient is, the more unfavorable the prognosis is, and this is probably related to late diagnosis, advanced clinical stage at onset, and a higher incidence of unfavorable histotypes (high-grade, poorly differentiated subtypes) [1, 2]. The development of CRC in children raises the suspicion of a genetic basis for the disease. Pediatric CRC patients are usually related to familial polyposis or ulcerative colitis [2, 3]. We recently treated a 10-year-old child for signet ring cell carcinoma of the colon, and this child had no familial polyposis or chronic ulcerative colitis. Moreover, we explored whether adenomatous polyposis coli gene *(APC*) mutations were the predisposing cause of CRC. The aim of this study was to recognize the spectrum of small mutations in the *APC* gene.

## Methods

This patient’s CRC tissues were collected from specimens by surgical resection at the Division of Gastroenterology and General Surgery, Department of Surgery, Kaohsiung Medical University Hospital. Written informed consent was obtained prior to the use of the resected specimen. Tissue samples were prepared utilizing standard formalin fixation, resulting in formalin-fixed paraffin-embedded (FFPE) tissue Immunohistochemistry was performed on unstained FFPE tissue.

### Genomic DNA extraction and PCR primer design

*APC*, the mutation cluster region (codons 1254–1631) on exon 15 was analyzed as described previously [4]. Genomic DNA was isolated from blood and tissue sample using Topgen total DNA Isolation Kit (Topgen Biotech, Taiwan) according to the manufacturer’s instructions.

Making use of the Human Genome build (NM_000038.5), M13-tailed PCR primers were designed by Primer Expression 3.0 (Applied Biosystems, USA) based on human reference genome (Chr.5 Sequence: nc000005.10), and primers were optimized for 100% coverage of the *APC* gene’s exon 15 coding sequences (Table 1). The *APC* gene comprises 15 exons, with exon 15 accounting for 77% of the coding sequence. In order to scan the exon 15 for mutations, the exon 15 was divided into 12 amplicons using specific primer pairs (Table 1).

**Table 1:** PCR primers, internal sequencing primers used for sequencing reactions and annealing temperatures for the amplification of each amplicon of *APC* gene

Amplicon	Primer name	Primer sequence	Size (bp)	PCR condition (see Table 2)
APC-E15.1 (with M13 tail)	APC-E15.1 forward	5′- TGTAAAACGACGGCCAGTTGTGACCTTAATTTTGTGATCTCTTGAT -3′	634	A
	APC-E15.1 reverse	5′- CAGGAAACAGCTATGACCCCAAACTTCTATCTTTTTCAGAACGA -3′		
APC-E15.2 (with M13 tail)	APC-E15.2 forward	5′- TGTAAAACGACGGCCAGTCCCAGCTCCTCTTCATCAAGA -3′	668	A
	APC-E15.2 reverse	5′- CAGGAAACAGCTATGACCTGTTTGGGTCTTGCCCATCTT -3′		
APC-E15.3 (with M13 tail)	APC-E15.3 forward	5′- TGTAAAACGACGGCCAGTCAGATGAGCAGTTGAACTCTGGAA -3′	756	A
	APC-E15.3 reverse	5′- CAGGAAACAGCTATGACCCAGCTGATGACAAAGATGATAATGAAC -3′		
APC-E15.4 (with M13 tail)	APC-E15.4 forward	5′- TGTAAAACGACGGCCAGTCCACTTGCAAAGTTTCTTCTATTAACC -3′	708	A
	APC-E15.4 reverse	5′- CAGGAAACAGCTATGACCGAAGAACCTGGACCCTCTGAACT -3′		
APC-E15.5 (with M13 tail)	APC-E15.5 forward	5′- TGTAAAACGACGGCCAGTAATAAAGCACCTACTGCTGAAAAGAGA -3′	751	A
	APC-E15.5 reverse	5′- CAGGAAACAGCTATGACCTTTTTCCTCCTTGAGCCTCATC -3′		
APC-E15.6 (with M13 tail)	APC-E15.6 forward	5′- TGTAAAACGACGGCCAGTTCGAATCCCCTCCAAATGAG -3′	560	A
	APC-E15.6 reverse	5′- CAGGAAACAGCTATGACCAGGCGTGTAATGATGAGGTGAA -3′		
APC-E15.7 (with M13 tail)	APC-E15.7 forward	5′- TGTAAAACGACGGCCAGTTGATAAGCTCCCAAATAATGAAGATAGAG -3′	659	A
	APC-E15.7 reverse	5′- CAGGAAACAGCTATGACCTTATACATTCCTGCAACAGGTCATC -3′		
APC-E15.8 (with M13 tail)	APC-E15.8 forward	5′- TGTAAAACGACGGCCAGTTGTTGAAGATACCCCAGTTTGTT -3′	650	A
	APC-E15.8 reverse	5′- CAGGAAACAGCTATGACCTTGTCCTGCCTCGAGAGATT -3′		
APC-E15.9 (with M13 tail)	APC-E15.9 forward	5′- TGTAAAACGACGGCCAGTCAGGGGAGAAAAGTACATTGGA -3′	692	B
	APC-E15.9 reverse	5′- CAGGAAACAGCTATGACCTCCTTTGGAGGCAGACTCAC -3′		
APC-E15.10 (with M13 tail)	APC-E15.10 forward	5′- TGTAAAACGACGGCCAGTCAGATGAGCCAACAGAACCTT -3′	632	A
	APC-E15.10 reverse	5′- CAGGAAACAGCTATGACCTCACTGGATTCTGATGAAGCA -3′		
APC-E15.11 (with M13 tail)	APC-E15.11 forward	5′- TGTAAAACGACGGCCAGTCGTGAGCACAGCAAACATTC -3′	663	B
	APC-E15.11 reverse	5′- CAGGAAACAGCTATGACCTTTGCTTGAGCTGCTAGAACTG -3′		
APC-E15.12 (with M13 tail)	APC-E15.12 forward	5′- TGTAAAACGACGGCCAGTCCCCTGACCAAAAAGGAACT -3′	623	B
	APC-E15.12 reverse	5′**-** CAGGAAACAGCTATGACCGAAGTTGGGATGGGATGCTA **-**3′		

### Amplification reactions and conditions

To enable a fast sequencing approach, the amplifications were performed using 50 ng /μl of the extracted DNA with the Topgen Fast PCR Master Mix (Topgen Biotech) on StepOne Real-Time PCR system (Applied Biosystems). The structure of the *APC* gene sequence did not permit the use of a single optimal thermal profile for all amplification primers, so 2 amplification conditions were designed to fit the primer sequences and amplicon lengths (Tables 1 and 2). For each of the amplified products, 2 μL was analyzed by agarose gel electrophoresis to check the amplification quality and quantity.

**Table 2:** PCR mixes and cycling conditions

PCR condition	PCR mix					Cycling conditions			Cycle number
	PCR Master Mix	Forward primer (1 μM)	Reverse primer (1 μM)	DNA (50 ng/μl)	Final volume	Denaturation	Annealing	Extension	
A	10 μL	4 μL	4 μL	2 μL	20 μL	96 °C	58 °C	74 °C	40
						30 SEC	30 SEC	30 SEC	
B	10 μL	4 μL	4 μL	2 μL	20 μL	96 °C	60 °C	68 °C	35
						15 SEC	15 SEC	15 SEC	

### DNA sequencing and sequence alignment

Each amplicon was sequenced in both forward and reverse directions using each M13-tailed forward and reverse primers (Table 1). Sequencing reactions were performed on 3130xl Genetic Analyzer following standard sequencing protocol (Applied Biosystems, USA). The sequence results for each sample were analyzed using CLC Genomics Workbench (CLC bio, USA) to verify the results and to identify putative mutations in each sample.

### *KRAS, NRAS* and *BRAF* molecular analysis

The specimens consisted of formalin-fixed, paraffin-embedded (FFPE) colorectal adenocarcinomas, and these specimens were submitted for clinical *KRAS*, *NRAS* and *BRAF* mutational analysis. FFPE samples were deparaffinized and air-dried, and DNA was subsequently isolated through direct DNA sequencing and high-resolution melting (HRM) analysis using the proteinase K and QIAamp microDNA extraction kit (QIAGEN GmbH, Hilden, Germany) [5].

### IHC of MMR protein expression

Immunohistochemistry was performed using the standard streptavidin-biotin- peroxidase procedure on the FFPE colorectal adenocarcinoma tissue [6]. Sections of thickness 4 μm were serially cut from the FFPE tissue blocks of each patient’s sample. The slides were deparaffinized in two changes of xylenes, rehydrated with graded alcohols, and then washed in tap water. Antigen retrieval was performed using Target Retrieval Solution with a pH of 9.0 (DAKO, Glostrup, Denmark). Endogenous peroxidase in the section was blocked by incubation in 3% hydrogen peroxide. The sections were incubated with a polyclonal antibody. The DAKO REAL EnVision Detection System-HRP (DAKO, Glostrup, Denmark) was then applied. Finally, sections were incubated in 3′,3-diaminobenzidine, and Mayer’s hematoxylin counterstaining was performed. Dehydration was achieved through two changes of 95% ethanol and two changes of 100% ethanol. The samples were cleared in three changes of xylene and then mounted. Whole tissue sections were interpreted by our pathologist who was blinded to the patient’s clinical characteristics.

Tumors with a total absence of nuclear staining but whose adjacent lymphocytes and/or nonneoplastic epithelial or stromal cells had any nuclear staining were scored ‘negative’ for expression of that protein. Expression was considered positive if the reverse was true. Therefore, loss of MMR expression was defined as the absence of detectable tumor cell nuclear staining in the presence of nuclear staining in adjacent lymphocytes and/or in nonneoplastic epithelial or stromal cells, which served as internal positive controls.

### Tumor suppressor gene (TP53)

Screening for TP53 mutations was conducted by Sanger sequencing. Genomic DNA from blood and tissue was extracted with the total DNA extraction kit (Topgen Biotech, TW). Exon-specific primer set for TP53 exons 2–10 were designed and synthesized by Topgen Biotech TW (Table 3). PCR were performed by 2X TaqPlus PCR Master Mix (Topgen Bioteh, TW) using the following program 95 °C PCR 5 min, 35 cycles for 95 °C 15 s, 60 °C 30 s 72 °C 30 s and final 72 °C 2 min then 25 °C 30 s on ABI Veriti PCR system. All the PCR products were purified by DNA column (Topgen Biotech, TW) and then sequenced with BigDye Terminator version 3.1 Cycle Sequencing kit (Applied Biosystems) and analyzed with a 3730xl ABI capillary electrophoresis system. Forward strand and reverse strand sequencing results were aligned with the reference of TP53 coding sequence (NM_001126114) and confirmation of TP53 mutations were also check with COSMIC Database (cancer.sanger.ac.uk/cosmic).

**Table 3:** Exon-specific primer set for *TP53* exons 2–10

TP53 Primer Set	Sequence (5′-3′)	Position (Ref: NC_000017.11 GRCh38.p7)	Exon Coverage	Amplicon length (bp)
TP53_I1–2-732 bp-E234-F TP53_I4–5-732 bp-E234-R	AGGGTTGGAAGTGTCTCATGCT GGGGGATACGGCCAGG	7676668 7675937	2, 3, 4,	732
TP53_I4–5-518 bp-E56-F TP53_I6–7-518 bp-E56-R	CTGCCGTCTTCCAGTTGCTT CACCTGGAGGGCCACTGA	7675310 7674793	5, 6	518
TP53_I6–7-719 bp-E789-F TP53_I9–10-719 bp-E789-R	CCTGCTTGCCACAGGTCTC AAAAGTGAATCTGAGGCATAACTGC	7674353 7673635	7,,8, 9	719
TP53_I8–9-547 bp-E910-F TP53_I10–11-547 bp-E910-R	CAGGACAAGAAGCGGTGGA GCAGGCTAGGCTAAGCTATGATGT	7673691 7673145	9, 10	547

## Case presentation

A 10-year-old Taiwanese boy was admitted to our hospital because of blood-tinged stool and chronic abdominal pain. He was healthy until a few months before admission when he complained of intermittent, colicky abdominal pain, constipation and abdominal fullness in addition to exhibiting body weight loss. Medical or congenital as well as family histories were unremarkable. We performed colonoscopy examinations on the patient’s parents and sibling, which revealed no colonic polyp. Physical examination revealed abdominal distention. He exhibited an enlarged, palpable right upper quadrant abdominal mass, and his stool contained bright red blood. Laboratory tests indicated a normal serum carcinoembryonic antigen (CEA) level. Past medical and familial histories were unremarkable for colonic malignancy.

**Fig. 1:**
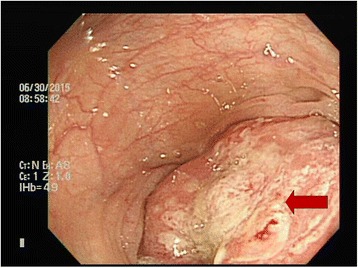
Colonoscopy revealed an approximately 4/5 circumferential obstructing ulcerative tumor in the proximal transverse colon near the hepatic flexure

**Fig. 2:**
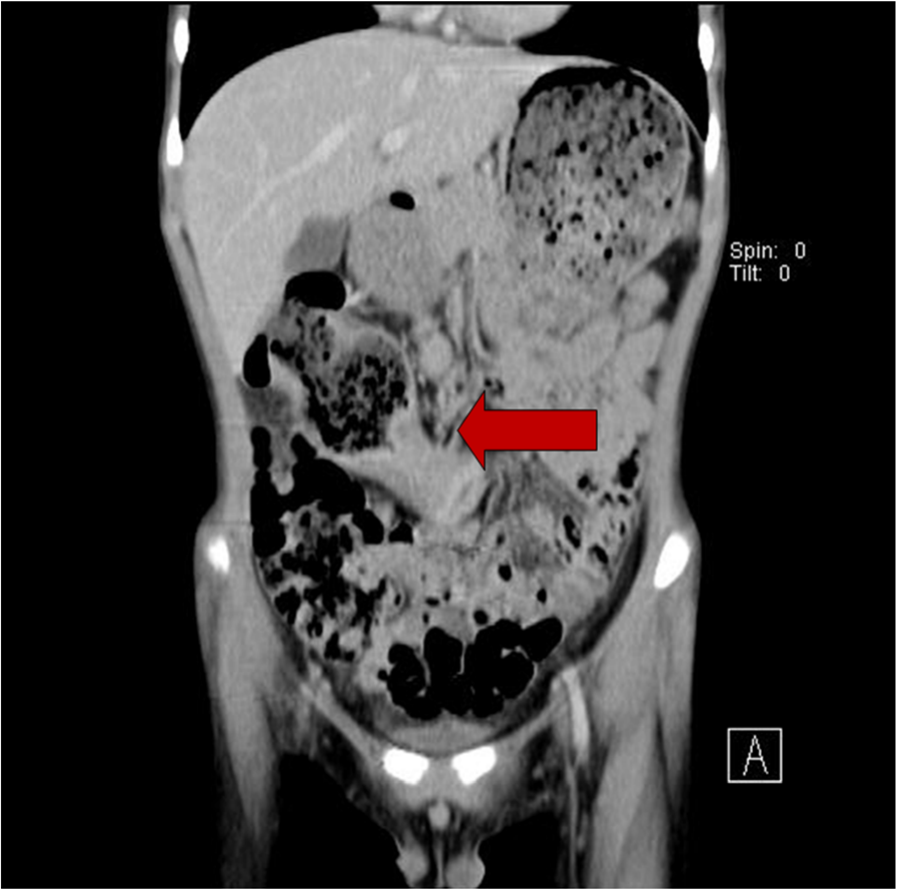
Contrast-enhanced computed tomography (CT) scan of the abdomen and pelvis showed annular wall thickening in the transverse colon with mild pericolic fat infiltration and visible clustered lymph nodes in the adjacent mesenteric space, compatible with transverse colon cancer

Colonoscopy revealed a nearly 4/5 circumferential obstructing ulcerative tumor in the proximal transverse colon near the hepatic flexure, and pathological examination involving colonoscopic biopsy showed signet ring cell carcinoma (Fig. 1). A contrast-enhanced computed tomography (CT) scan of the abdomen and pelvis revealed annular wall thickening in the transverse colon with mild pericolic fat infiltration and visible clustered lymph nodes in the adjacent mesenteric space, compatible with transverse colon cancer (Fig. 2).

First, a loop ileostomy was performed to divert stool from the ileum. Exploratory laparotomy was performed 1 week later, detecting a tumor near the hepatic flexure in the wall of the transverse colon. A radical extensive right hemicolectomy was performed, and a segment of the mesentery including the vessels draining this area was resected. The liver, terminal ileum, and peritoneum were normal to palpation. The resected specimen contained a firm, sessile, ulcerative tumor 4.0 cm long and 5.3 cm wide. On the basis of the aforementioned imaging and pathology results, the patient was finally diagnosed with grade 3, UICC stage IIIB (T3N2bM0), and poorly differentiated transverse colon cancer and signet ring cell carcinoma with lymph-vascular and perineural invasions.

Genetic features of the postoperative surgical specimen revealed wild-type *BRAF* without mutation in codon 600, wild-type *KRAS* without mutation in codons 12, 13, 61, and 146, and wild-type *NRAS* without mutation in codons 12, 13, 59, 61, 117, and 146. We collected tumor samples and subjected them to an immunohistochemistry (IHC) test, and the results revealed no loss of nuclear expression of mismatch repair (MMR) proteins, including MLH1, MSH2, MSH6 and PMS2, indicating a low probability of tumors with high microsatellite instability (MSIH). Otherwise, screening for *TP53* mutations found that blood DNA and tissue DNA own the same genetic mutation *TP53* c.215C > G.

**Fig. 3:**
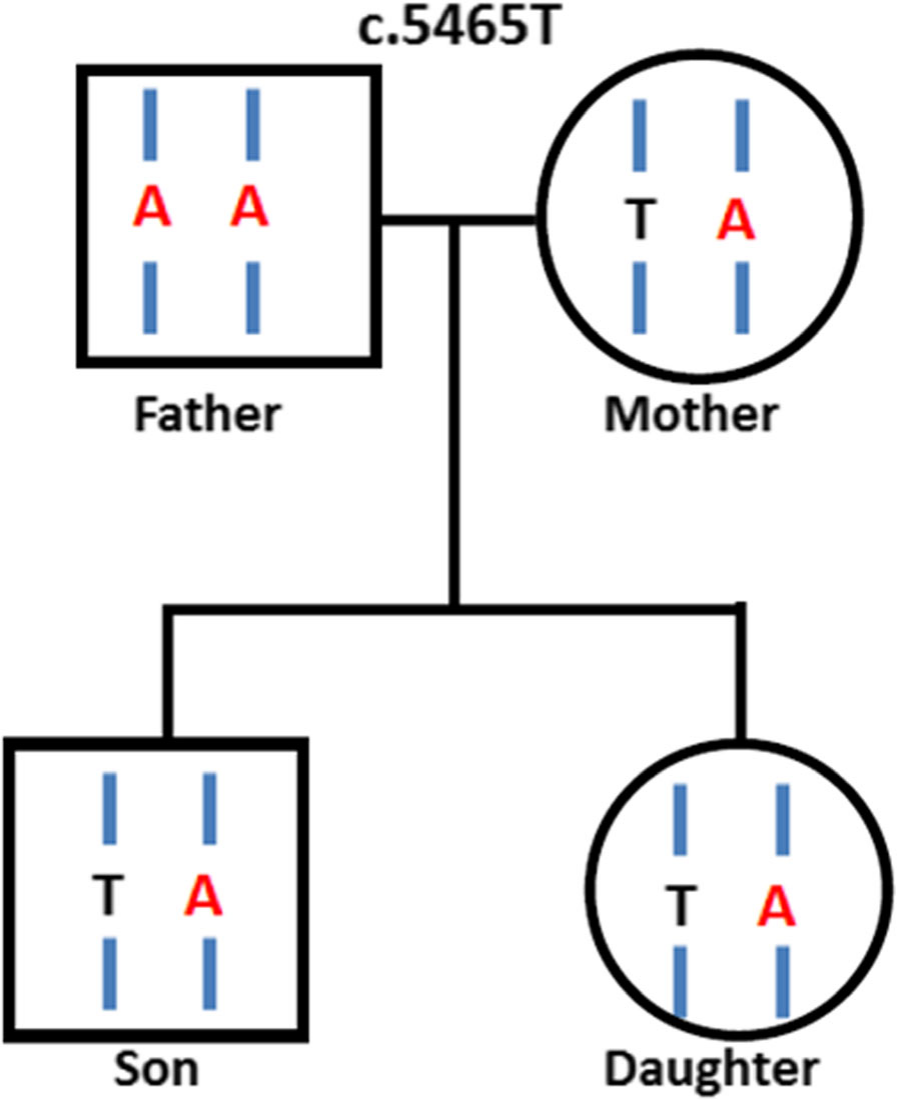
Family pedigree shows that the patient’s father carried a homozygous germline mutation in exon 15 of *APC* at c.5465 T > A, whereas his mother and sister carried heterozygous germline mutation. The patient carried heterozygous germline and heterozygous somatic (c.7397C > T) mutations in both blood and tissue samples

Notably, we identified a missense mutation (point mutation) in exon 15 of *APC*. In addition, we determined a heterozygous germline mutation at c.5465 T > A and a heterozygous somatic mutation at c.7397C > T. These both germline and somatic mutations may be the predisposing cause of CRC. The family pedigree for the *APC* germline mutation at c.5465 T > A was derived using blood samples (Fig. 3).

After operation, we administered a regimen of modified FOLFOX-6 to the patient as adjuvant chemotherapy. No chemotherapy-related grade 3 or higher toxicities were noted in the first six treatment cycles. Chemotherapy was tolerated adequately with excellent performance along with the normalization of all liver enzymes, and the absence of distant metastases, confirmed through CT, was considered to indicate disease stabilization. In total 12 cycles of modified FOLFOX-6 were administrated to the patient, and he was followed-up closely at our clinic (Table 4).

**Table 4:** Summary of information from this case report

Date	Information
June 2015	Abdominal CT: annular wall thickening in the transverse colon with mild pericolic fat infiltration and visible clustered lymph nodes in the adjacent mesenteric space
June 2015	Colonoscopy: nearly 4/5 obstructing ulcerative tumor in the proximal transverse colon
July 2015	Transverse loop-ileostomy for stool diverting; followed by extended right hemicolectomy
July 2015	Pathology: signet ring cell carcinoma, grade 3; UICC stage IIIB (T3N2bM0)
July 2015	Genetic features: biallelic germline and somatic mutations in *APC* gene
August 2015	Modified FOLFOX-6 were administrated to the patient
December 2015	Abdominal CT: no distant metastatic lesion or recurrent mass
June 2016	Colonoscopy: no recurrent tumor found

## Discussion

CRC is one of the leading causes of cancer mortality in adults; however, it is extremely rare in the pediatric age group. Less than 1% of all malignant growths of the large bowel occur in people aged younger than 20 years. The reported peak age is 15 years old, whereas the youngest reported patient is a 9-month-old female infant [7]. However, because of a lack of awareness of this rare disease, diagnosis is usually delayed until the disease reaches an advanced stage, resulting in an extremely poor prognosis in children compared with that in adults [8, 9].

Abdominal pain and vomiting were the most common symptoms in these cases. However, these symptoms are nonspecific in children because the symptoms may mimic several common functional gastrointestinal disorders [9]. In children, there are several other causes of abdominal pain that are considerably more common than carcinoma of the colon [8]. In not-at-risk patients, early diagnosis is difficult because of a low level of suspicion for pediatric colon cancer; therefore, its presentation is typically at an advanced stage with up to 60% of children exhibiting luminal obstruction, whereas only 18% of adults show similar symptoms [10]. The fecal occult blood test is a simple noninvasive but nonspecific procedure, and, if positive, it should arouse suspicion of bowel pathology, necessitating further investigation such as colonoscopy and abdominal CT. Otherwise, the serum CEA level can be used to determine the recurrence of tumors, which is indicated by a postoperative decrease followed by a gradual increase in titers [8, 10]. Here, we report the first confirmed case of a 10-year-old Taiwanese boy with CRC caused by both germline and somatic mutations in the *APC* gene.

A genetic analysis of the postoperative surgical specimen revealed wild-type *BRAF* without mutation in codon 600, wild-type *KRAS* without mutation in codons 12, 13, 61, and 146, and wild-type *NRAS* without mutation in codons 12, 13, 59, 61, 117, and 146. Subjecting the tumor samples to the IHC test revealed no loss of nuclear expression of MMR proteins, including MLH1, MSH2, MSH6, and PMS2, indicating a low probability of MSIH tumors. The pedigree of familial colon cancer provided insufficient evidence.

Analysis of MMR protein expression using IHC is an acceptable alternative test that identifies the affected gene by detecting loss of its protein product. The test is widely available and does not require the use of a molecular laboratory. IHC-detected loss of MMR protein expression was demonstrated to be highly concordant with DNA-based MSI testing and has good sensitivity (>90%) and excellent specificity (100%) [11]. Both the germline mutation at c.5465 T > A and the somatic mutation at c.7397C > T are missense mutations of the *APC* gene, and we have identified c.215C > G germline mutation are missense mutations of the *TP53* gene. One study reported that other tumor suppressors resulting in MSS tumors, such as MutYH, PolD, PolE, and NTHL1, might be associated with the pathogenesis of CRC in addition to *APC* gene mutations.

Genetic factors were undoubtedly involved in the development of colon cancer in our patient at the age of 10 years. We suspected that a mutation increased the risk of colon cancer, as demonstrated in the *APC* mutation analysis. The patient’s father carried a homozygous germline mutation in exon 15 of *APC* at c.5465 T > A, whereas his mother and sister carried a heterozygous germline mutation. Moreover, we detected missense mutations in exon 15 of *APC* including a heterozygous germline mutation at c.5465 T > A and a heterozygous somatic mutation at c.7397C > T. Either of the germline mutation or the somatic mutation may have caused the cancer development.

For the rare first-decade CRC patient carrying both germline and somatic mutations in the *APC* gene in addition to multiple polyposis, the pathogenesis of the CRC may have had a distinct genetic component. According to our review of the literature, this is the first study revealing the presence of both germline and somatic mutations in a child. Because of this rare finding of a gene mutation in a child with colon cancer, we conclude that it may be one of the predisposing causes of pediatric CRC. Therefore, screening patients with early-onset CRC through clinical and genetic characterizations is essential.

*TP53* gene mutations will contribute to the understanding of gene-environment interactions in cancer, in particular when comparing variations in *TP53* mutation in relation to different cohorts of patients [12, 13]. Therefore, further studies are mandatory to determine that *TP53* gene mutations modulate their impact on cancer development and prognosis in early-onset CRC and this will elucidate the P72R germline variant segregate within the family pedigree.

The clinical outcomes of our patient must be carefully followed up because delayed diagnosis, advanced stages of disease at presentation, and, particularly, poor differentiation in histology examinations are the major determinants of unsatisfactory outcomes.

## Conclusions

A possibility of colonic carcinoma in children should not be excluded only on the basis of age. The reported case is an example demonstrating the necessity of conducting additional studies to assess all factors determining the risk of pediatric CRC and to clarify the underlying genetic mechanism for each factor.

## Abbreviations

*APC*: Adenomatous polyposis coli gene
*BRAF*: B-type Raf kinase
CEA: Carcinoembryonic antigen
CRC: Colorectal carcinoma
CT: Computed tomography
FFPE: Formalin-fixed paraffin-embedded
IHC: Immunohistochemistry
*KRAS*: Kirsten Rat Sarcoma Viral Oncogene Homolog
MLH1: MutL homolog 1
MLH2: MutL homolog 2
MMR protein: Mismatch repair protein
MSH6: MutS homolog 6
MSIH: microsatellite instability
*NRAS*: Neuroblastoma RAS viral oncogene homolog
PMS2: Postmeiotic segregation increased 2
UICC: Union for International Cancer Control
